# Diabetes in pregnancy: a new decade of challenges ahead

**DOI:** 10.1007/s00125-018-4545-y

**Published:** 2018-01-22

**Authors:** Ute Schaefer-Graf, Angela Napoli, Christopher J. Nolan, the Diabetic Pregnancy Study Group

**Affiliations:** 1 Berlin Center for Diabetes in Pregnancy, Department of Obstetrics and Gynecology, St Joseph’s Hospital, Wüsthoffstraße 15, 12101 Berlin, Germany; 2 Department of Obstetrics, Charité, Humboldt University, Berlin, Germany; 3 Department of Clinical and Molecular Medicine, Sant’Andrea Hospital, Faculty of Medicine and Psychology, Sapienza University, Rome, Italy; 4 Department of Endocrinology, The Canberra Hospital, Garran, ACT Australia; 5 Australian National University Medical School and John Curtin School of Medical Research, Australian National University, Acton, ACT Australia

**Keywords:** Fetus, Gestational diabetes mellitus, Obesity, Placenta, Pregnancy, Review, Type 1 diabetes, Type 2 diabetes

## Abstract

Every 10 years, the Diabetic Pregnancy Study Group, a study group of the EASD, conducts an audit meeting to review the achievements of the preceding decade and to set the directions for research and clinical practice improvements for the next decade. The most recent meeting focused on the following areas: improving pregnancy outcomes for women with pregestational type 1 diabetes and type 2 diabetes; the influence of obesity and gestational diabetes on pregnancy outcomes; the determinants and assessment of fetal growth and development; and public health issues, including consideration of transgenerational consequences and economic burden. The audit meeting also considered the likely impact of ‘omics’ on research within the field and the potential of these technologies to enable precision-medicine approaches to management. Through sharing of the findings and ideas of audit meeting participants, the DPSG hopes to promote networking, research and advances in clinical care, to improve outcomes for all women and their offspring affected by diabetes and obesity in pregnancy.

## Introduction

The Diabetic Pregnancy Study Group (DPSG) (www.dpsghome.org) of the EASD aims to further research and promote education to improve the management of pregnant women with diabetes and their offspring. Every 10 years, DPSG conducts an audit meeting to review the achievements of the previous decade and to set the directions for research and clinical practice improvements for the next decade. This review is a summary report of the October 2016 audit meeting sessions held in Dublin, Ireland. The challenges surrounding diabetes in pregnancy, facing researchers and clinicians over the coming decade, are summarised in the Text box.
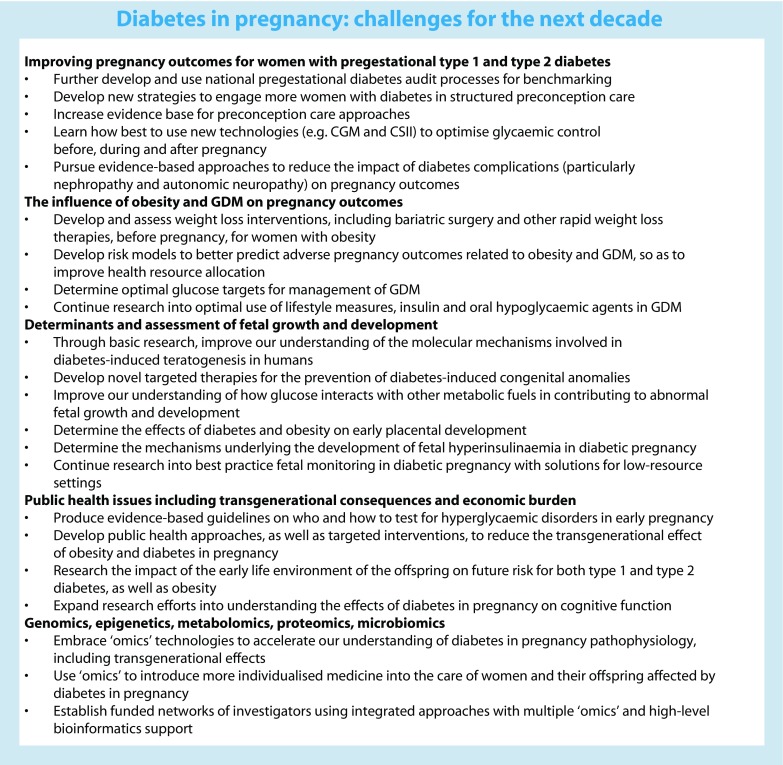


## Improving pregnancy outcomes for women with pregestational type 1 and type 2 diabetes

Women with pregestational type 1 and type 2 diabetes mellitus continue to have poorer pregnancy outcomes than the background population, including a three- to fourfold higher rate of perinatal mortality [1, 2]. However, lower stillbirth rates have recently been reported in centres involved in the UK National Pregnancy in Diabetes (NPID) 2015 audit compared with those reported in the Confidential Enquiry into Maternal and Child Health audit from 2002–2003, suggesting that improvement is possible and highlighting the value of national audit programmes [3]. Importantly, social disadvantage was still strongly related to poorer diabetic pregnancy outcomes in the NPID audit [3]. The challenges ahead include increasing the percentage of women with diabetes who prepare for pregnancy, translating new glycaemic control technologies to the circumstances of pregnancy and reducing the impact of diabetes complications on maternal and fetal outcomes.

### Preconception care

The available evidence strongly suggests that structured preconception care for women with pregestational diabetes reduces the risk of major congenital anomalies and perinatal mortality in women with type 1 and type 2 diabetes and is cost-effective [4, 5]. This care encompasses optimisation of glycaemic control, assessment and management of diabetes complications, cessation of potentially harmful drugs and commencement of folic acid, delivered by an experienced multidisciplinary team. Local and national campaigns have not achieved sufficient rates of pregnancy preparation in women with pregestational diabetes [6]. It remains unclear how best to improve these results. Healthcare teams need to promote awareness of preparation for a successful pregnancy to all adolescent girls and women of child-bearing age who have diabetes. Confidential and expert counselling on sexual and reproductive health, including advice on use of contraception and avoidance of unplanned pregnancies and unsafe sexual practices, is essential.

Due to the lack of high-level RCT evidence in this area [7], studies trialling different preconception care approaches in women with pregestational diabetes are being encouraged and a core outcome set has been developed for this purpose [8].

### Optimising glycaemic control

The well-known harmful consequences of maternal hyperglycaemia must be balanced against the significant risk of hypoglycaemia, despite lack of data on the effects of maternal hypoglycaemia on neonatal outcomes [9]. The importance of optimal control in the first and second trimesters for prevention of pre-eclampsia, preterm birth and large for gestational age (LGA) neonates is becoming clearer [3, 10]. The American Diabetes Association and the UK National Institute for Health and Care Excellence targets for glycaemic control for women with type 1 diabetes during early pregnancy (HbA_1c_ <48 mmol/mol [<6.5%]), individualised for safety, seem to be reasonable [11, 12]. Based on the recent NPID audit results, HbA_1c_ levels of <42 mmol/mol (<6.0%) in later pregnancy should be safely achievable without serious hypoglycaemia in some women with type 1 diabetes and many women with type 2 diabetes [3].

Engagement of women in their own glycaemic control through diabetes education, with promotion of sensible lifestyle, frequent self-monitoring of blood glucose and a supported active approach to insulin adjustment is clearly important. While the use of insulin analogues (e.g. lispro, aspart, glargine and detemir) can be associated with reduced hypoglycaemia and glucose excursions, the safety and efficacy of newer insulin analogues and concentrated insulin preparations need to be clarified. Improved pregnancy outcomes from the use of continuous subcutaneous insulin infusion (CSII) have not yet been shown, such that its use should be on a case-by-case basis [13].

A recent RCT comparing continuous glucose monitoring (CGM) with capillary blood glucose monitoring only in women with type 1 diabetes (CONCEPTT) showed that CGM during pregnancy can increase the percentage of time that blood glucose is in the target range and reduce neonatal complications [14]. CGM compared with capillary blood glucose monitoring resulted in an approximately 50% reduction in LGA, neonatal intensive care admissions >24 h and neonatal hypoglycaemia [14].

Combining CGM and CSII systems, with or without closed-loop, may enable more effective insulin pump use in pregnancy, with studies in selected populations showing promise [15, 16].

### Diabetes complications

Retinopathy, nephropathy and neuropathy frequently affect pregnancies of women with pregestational diabetes. Less common, but potentially life-threatening, is ischaemic heart disease. Guidelines recommend screening for diabetes complications before conception, during gestation and after delivery, as they can manifest or progress at these times [12, 13]. Diabetic nephropathy is associated with higher rates of congenital anomalies and pre-eclampsia [17, 18]. We would encourage study of pre-eclampsia prevention in diabetic women, with and without nephropathy, through the exploration of early biological and clinical markers and the use of low-dose aspirin [19]. The impact of lower blood pressure targets on pregnancy outcomes, including pre-eclampsia rates [17], and the effect of intravitreal dexamethasone implants on diabetic retinopathy during pregnancy [20] are topics that warrant further research. Improved management approaches to women with autonomic neuropathy causing gastroparesis and/or postural hypotension are required.

## The influence of obesity and gestational diabetes mellitus on pregnancy outcomes

### Obesity and gestational diabetes mellitus

Obesity in women of reproductive age is increasing worldwide and its negative impact on various pregnancy outcomes, including the risk of gestational diabetes mellitus (GDM), is now realised [21]. The 2009 Institute of Medicine guidelines on gestational weight gain (GWG) recommend that women with BMI ≥30 kg/m^2^ gain 5–9 kg during pregnancy. While there is evidence to support not exceeding these GWG recommendations, whether to recommend a GWG of <5 kg remains debatable due to an increased risk of small for gestational age (SGA) neonates [22, 23]. For women with BMI ≥35 kg/m^2^, however, a gestational weight change in the range of −4.9 to +4.9 kg has been reported to reduce the incidence of LGA births without an increase in SGA births [23].

As for bariatric surgery, retrospective studies show that it improves fertility, reduces the incidence of GDM, pre-eclampsia and LGA births, but increases rates of SGA births and possibly maternal venous thromboembolism and perinatal mortality [24–26]. Well-conducted prospective studies of bariatric surgery pre-pregnancy are required. In addition, research into the alternatives to bariatric surgery for rapid weight loss before and between pregnancies, such as very low energy diets, pharmacological therapy (e.g. liraglutide) and endoluminal devices is warranted.

### Prevention of GDM

To date, interventions to prevent GDM in at-risk women have generally not been successful. Despite the known adverse effects of excessive GWG in obese women [27], multiple RCTs of lifestyle interventions during pregnancy have largely failed to prevent GDM, even though some reduction in GWG can be achieved [28, 29]. Whether such interventions need to commence earlier [30], have a greater effect on limiting GWG or work only in certain phenotypes or genotypes [31] is unknown. Despite hope from small studies, use of vitamin D, fish oil, metformin and inositol for GDM prevention have not been confirmed in larger RCTs [32–34]. We await results of larger probiotics studies, such as the Study of Probiotics in the Prevention of Gestational Diabetes (SPRING) trial [35]. Failure of strategies to prevent GDM could be a consequence of established dysfunction of glucose regulation, particularly of islet beta cells, well in advance of the affected pregnancy. Focus on GDM prevention is now turning to lifestyle interventions before and between pregnancies [36].

### Predicting risk in GDM and treatment approaches including glucose targets

Due to the high prevalence of GDM, there is increasing interest in risk stratification to more effectively allocate limited healthcare resources. Risk engines are being developed (e.g. Brisbane risk model, unpublished, H. D. McIntyre, Mater Health Services and The University of Queensland, South Brisbane, QLD, Australia), recognising the fact that blood glucose needs to be considered in the context of other factors, such as maternal age and BMI. Another tool for risk stratification is fetal biometry, with tight vs less-tight glycaemic control being advocated for fetuses with greater or normal abdominal circumferences, respectively [37].

Ongoing controversies in GDM include whether early diagnosis is useful, glucose targets for management and the role for oral hypoglycaemic agents. Of note, glucose levels in healthy pregnant women are lower than recommended target levels in GDM guidelines [38], and both fasting and postprandial glucose levels in GDM adversely affect outcomes [39]. However, too-aggressive glucose lowering in certain women could result in an SGA birth, with the potential for detrimental long-term effects in the offspring. Of the oral glucose-lowering agents metformin shows most promise but its use in pregnancy is debatable due to its easy placental transfer [40].

## Determinants and assessment of fetal growth and development

The most serious adverse outcomes of diabetic pregnancy continue to be congenital anomalies, stillbirth and excessive fetal growth. Our understanding of the causative mechanisms, however, remain limited. Improved accuracy of fetal growth assessment is essential to guide obstetric management.

### Congenital anomalies

Poor glycaemic control at the time of conception and during the first trimester is clearly linked to higher rates of congenital anomalies. Multiple non-glycaemic factors (e.g. cytokines and other nutrients) interacting with glucose and the fetal genotype are also likely to influence teratogenesis. Within rodent models, oxidative, hexosamine and endoplasmic reticulum stresses, as well as disturbed autophagy and apoptosis processes, have all been implicated [41, 42]. Greater focus on determining human diabetes-induced teratogenic pathways is required; this might involve the use of human embryonic stem cells [43]. The long-term goal is development of novel targeted therapies to prevent diabetes-induced congenital anomalies.

### Maternal, placental and fetal determinants of fetal growth

While maternal blood glucose control and adiposity clearly contribute to excess fetal adiposity, fetal factors, including fetal sex, genes and the presence or absence of hyperinsulinaemia, are also important determinants of fetal growth [44–47]. Once established, fetal hyperinsulinaemia can potentially contribute to an exaggerated fetal glucose steal [44]. This hypothesis might explain the occurrence of macrosomia in pregnancies with (near) normal maternal glucose values in late pregnancy [44].

There is still much to be discovered about how maternal lipids and their placental handling contribute to neonatal adipose tissue development. Complex regulatory mechanisms involving peroxisome proliferator-activated receptors are involved [48, 49]. Neonatal adiposity correlates with maternal BMI and plasma triacylglycerol, although it negatively correlates with cord serum triacylglycerol, suggesting increased fetal tissue lipid uptake [46, 47]. Indeed, hyperinsulinism increases lipoprotein lipase activity, a putative mechanism leading to the increased incorporation of lipids into fetal adipocytes [45]. Deficiency in polyunsaturated fatty acids occurs in parallel with increased lipoperoxidation in maternal diabetes [50].

### Early pregnancy and diabetic fetopathy

Maternal first-trimester HbA_1c_ is a good predictor of fetal macrosomia [51]. In addition, dietary glycaemic index and glycaemic load in early, but not late, pregnancy has been linked with offspring childhood adiposity [52]. There is also evidence indicating that fetal hyperinsulinaemia develops in early pregnancy [44]. Thus, the effects of diabetes on early placental and fetal islet development [53–55], key determinants of fetal growth and development, need further investigation. Lifestyle and diabetes management intervention RCTs focused on conception and the first-trimester time-points are warranted.

### Assessment of fetal growth

Correct assessment of fetal growth prior to delivery is crucial for determining optimal management of delivery. A recent study suggests that fractional thigh volume is the best predictor of neonatal percentage body fat and birthweight *z* scores in suspected macrosomic fetuses [56]. MRI, while being more specific, is not more sensitive than two-dimensional ultrasound in detecting macrosomia [57] and its cost and availability are prohibitive. Assessments may be improved by the use of customised fetal growth charts, although they require further optimisation and need to be more inclusive of diverse populations [58]. Identification of novel biomarkers indicative of excessive fetal growth would be an advance. RCTs to determine optimal timing and frequency of ultrasound assessments in women with GDM are required, so that limited resources can be best allocated. Surveillance strategies also need to be developed for use in low-resource settings.

## Public health issues including consideration of transgenerational consequences and economic burden

Increasing prevalence of obesity and diabetes is placing a major burden on maternity health services in developed and developing countries. Diabetes in pregnancy is associated with increased long-term cardiometabolic risk for both women and offspring. Optimal management of GDM, obesity and pregestational diabetes in pregnancy has the potential to lessen the transgenerational and population-level impact of metabolic disease.

### Diagnosis of GDM

The WHO adopted the International Association of Diabetes in Pregnancy Study Groups (IADPSG) 2010 criteria for the diagnosis of GDM in 2013 [59, 60]. Many countries have adopted these diagnostic criteria, with some regional accommodations, but approaches to diagnosing GDM still vary widely. Performance of an OGTT during pregnancy after a screening glucose challenge test has not been validated against the IADPSG diagnostic approach. Diagnostic criteria for GDM have not been validated for early pregnancy, although early screening for ‘overt diabetes’ (IADPSG) or ‘diabetes in pregnancy’ (WHO) in at-risk women is recommended [61]. Further research to determine the value of testing for and treating early GDM (<24 weeks gestation), as well as optimal early diagnostic criteria, is required.

### Public health issues and GDM

GDM is an independent and robust risk factor for the progression of women and their offspring to cardiometabolic diseases [62]. Early identification of women and offspring at highest risk after delivery and development of evidence-based prevention programmes are required. Lifestyle change and metformin treatment, initiated on average 12 years following a GDM pregnancy, and followed for 10 years post intervention, have been demonstrated to be highly effective in reducing progression to type 2 diabetes [63]. There is insufficient data to show whether treating maternal GDM has any effect on the long-term metabolic health of offspring. This may be due to suboptimal maternal therapy and/or insufficient follow-up time, or to stronger influence of the post-delivery family lifestyle. Post-pregnancy intervention programmes for the offspring need to be developed.

### Type 1 diabetes and transgenerational issues

Type 1 diabetes occurs in genetically susceptible individuals and evidence of islet cell autoimmunity appears long before diagnosis [64]. Having a mother with type 1 diabetes is associated with a lower risk of future diabetes compared with the genetic/shared-environmental risk of having a father or sibling with type 1 diabetes [65]. The implications that the diabetic intrauterine endocrine–metabolic–immunological milieu might have on the conditioning of the developing endocrine pancreas is intriguing and is yet to be explored.

### Diabetes in pregnancy and cognitive disorders

Diabetes in pregnancy may have an impact on cognitive function and may increase the risk of autism spectrum disorders or attention deficit hyperactivity disorder in offspring [66]. However, lower cognitive test scores in offspring of women with GDM may be explained by co-existing risk factors rather than by maternal hyperglycaemia [66]. Of note, delayed cortical evoked responses in the neonatal period and at age 3 years were found in infants of mothers with type 1 diabetes [67]. More studies into the impact of maternal diabetes on neurocognitive development of offspring are required.

## Genomics, epigenetics, metabolomics, proteomics and microbiomics

The rapid emergence of the powerful ‘omics’ technologies, supported by bioinformatics, provides new insights into disease mechanisms, more accurate diagnosis and new precision therapies. The value of genomics for accurate diagnosis of neonatal diabetes and maturity-onset diabetes of the young is established [68]. New paradigms are evolving for precision-medicine approaches to type 2 diabetes, which will also be highly relevant to GDM and type 2 diabetes in pregnancy [68]. However, to date, genetic risk scores, derived from known diabetes-associated gene polymorphisms, have not meaningfully contributed to GDM prediction. Epigenetic changes have been proposed to be involved in the effects of in utero exposure to maternal hyperglycaemia on long-term metabolic health issues of the offspring [69]. An example of success using whole-transcriptome analysis comes from a study that showed marked upregulation of tryptophan hydroxylase-1 in islets of pregnant mice [70]. Serotonin is now an established mediator of the islet beta cell adaptive response to lactogenic hormones in pregnancy [70]. Proteomics is also being used to better understand islet beta cell responses to pregnancy [71]. Metabolomics profiles of fasting plasma in women with GDM at 6 weeks post-partum identified 21 amino acids and fatty acids that were able to distinguish those who would progress to type 2 diabetes [72]. While the maternal and offspring microbiome differ between GDM and normoglycaemic pregnancies, reproducible intervention trials with probiotics are still needed to translate these findings to clinical practice. Limitations need to be considered in ‘omics’ study design, as large cohorts are usually required for adequate power and ‘causation’ (as opposed to ‘association’) is often difficult to prove. Networking of investigators using integrated approaches with multiple ‘omics’ technologies and high-level bioinformatics support is recommended for future studies within our field.

## Conclusion

The work of the last decade has best defined the challenges ahead for research and clinical care in diabetes in pregnancy. Research and clinical focus on the preconception period and first trimester is essential to improve pregnancy outcomes for women with pregestational diabetes. New technologies for improving glycaemic control (CGM and CSII) in diabetic pregnancy show promise but it is still unclear how best to use them. The need to consider GDM within the context of other maternal characteristics, such as maternal ethnicity and BMI, has been realised. A reappraisal of whether GDM is a condition of late pregnancy only is required. The long-term maternal and offspring effects of any interventions for use in pregnancy are difficult to study, but need to be explored. Last, scientific endeavour within this field is central to achieving better outcomes for women with diabetes in pregnancy. This requires the development of networked programmes enabling the most effective use of powerful new research technologies and high-quality clinical trials for the translation of new discoveries into best practice.

## Appendix

### Attendees at DPSG audit meeting, Dublin, 2016

Writing group: Ute Schaefer-Graf^1^, Angela Napoli^2^ and Christopher J. Nolan^3^.

Contributing attendees: Eleni Anastasiou^4^, Katrien Benhalima^5^, Patrick Catalano^6^, Ana Chico^7^, Cheril Clarson^8^, Rosa Corcoy^7^, Donald R. Coustan^9^, Maria G. Dalfra^10^, Tine D. Clausen^11^, Peter Damm^12^, Philippe Deruelle^13^, Harold de Valk^14^, Gernot Desoye^15^, Roland Devlieger^16^, Josip Djelmis^17^, Anne Dornhorst^18^, Nicoletta Dozio^19^, Fidelma P Dunne^20^, Aoife M. Egan^20^, Ulf J. Eriksson^21^, Helena Fadl^22^, Denice S. Feig^23^, Sander Galjaard^24^, Emilio Herrera^25^, David Hill^8^, Alicia Jawerbaum^26^, Dorte M. Jensen^27^, Alexandra Kautzky-Willer^28^, Louise Kelstrup^12^, Annunziata Lapolla^10^, Jeannet Lauenborg^29^, Jacques Lepercq^30^, Robert S Lindsay^31^, Mary R. Loeken^32^, William L. Lowe^33^, Michael J. Maresh^34^, Elisabeth R. Mathiesen^12^, David R. McCance^35^, H. David McIntyre^36^, Giorgio Mello^37^, Sara J. Meltzer^38^, Boyd E. Metzger^39^, Helen R. Murphy^40^, Jenny E. Myers^41^, Yasue Omori^42^, Per Ovesen^43^, Martina Persson^44^, Maria del Pilar Ramos-Alvarez^25^, Kristina M. Renault^12^, Lene Ringholm^45^, Janet A. Rowan^46^, Luisa Ruas^47^, David A. Sacks^48^, Charles Savona-Ventura^49^, Eleanor Scott^50^, David Simmons^51^, Takashi Sugiyama^52^, Adam Tabak^53^, Mette Tanvig^54^, Rosemary C. Temple^55^, Anne Vambergue^56^, Mireille N.M. van Poppel^57^, Marta Viana^25^, Christina Vinter^54^, Ewa Wender-Ozegowska^58^, Parri Wentzel^21^, Agnieszka Zawiejska^58^ and Christos Zoupas^59^.

^1^Berlin Center for Diabetes in Pregnancy, Department of Obstetrics and Gynecology, St Joseph’s Hospital, Berlin, Germany

^2^Department of Clinical and Molecular Medicine, Sant’Andrea Hospital Faculty of Medicine and Psychology, Sapienza University, Rome, Italy

^3^Department of Endocrinology at The Canberra Hospital and the Australian National University Medical School, Canberra, ACT, Australia

^4^Diabetes Center, Alexandra General Hospital, Athens, Greece

^5^Department of Endocrinology, UZ Gasthuisberg, KU Leuven, Leuven, Belgium

^6^Centre for Reproductive Health, Department of Obstetrics and Gynecology, MetroHealth Medical Center, Cleveland, OH, USA

^7^Department of Endocrinology and Nutrition, Hospital de la Santa Creu I Sant Pau, Barcelona; CIBER-BBN, Zaragoza, Spain

^8^Lawson Health Research Institute, London, ON, Canada

^9^Division of Maternal-Fetal Medicine, Women & Infants Hospital of Rhode Island and Warren Alpert Medical School of Brown University, Providence, RI, USA

^10^Department of Medical and Surgical Sciences, University of Padua, Padova, Italy

^11^Department of Obstetrics and Gynaecology, Hilleroed Hospital, Hilleroed, Denmark

^12^Center for Pregnant Women with Diabetes, Department of Obstetrics, Copenhagen, Denmark

^13^Department of Obstetrics and Gynaecology, CHU Lille, Lille, France

^14^Department of Internal Medicine, University Medical Center Utrecht, Utrecht, the Netherlands

^15^Department of Obstetrics and Gynecology, Medical University of Graz, Graz, Austria

^16^Department of Obstetrics and Gynaecology, KULeuven, University of Leuven, Leuven, Belgium

^17^Department of Obstetrics and Gynecology, School of Medicine, Zagreb, Croatia

^18^Department of Endocrinology, Hammersmith Hospital, London, UK

^19^Department of Diabetes, Diabetes Research Institute, Ospedale San Raffaele and San Raffaele, Vita-Salute University, Milan, Italy

^20^School of Medicine, National University of Ireland, Galway, Ireland

^21^Department of Medical Cell Biology, Biomedical Center, Uppsala University, Uppsala, Sweden

^22^Department of Obstetrics and Gynecology, School of Health and Medical Sciences, Orebro University, Orebro, Sweden

^23^Department of Medicine, University of Toronto, Toronto, ON, Canada

^24^Department of Obstetrics and Gynaecology, Erasmus Medical Center (EMC) Rotterdam, Rotterdam, the Netherlands

^25^Facultad de Farmacia, Universidad, San Pablo-CEU, Madrid, Spain

^26^Laboratory of Reproduction and Metabolism, CEFYBO-CONICET, School of Medicine, University of Buenos Aires, Buenos Aires, Argentina

^27^Department of Endocrinology, Odense University Hospital, Faculty of Health Science, University of Southern Denmark, Odense, Denmark

^28^Internal Medicine III, Endocrinology & Metabolism, Gender Medicine Unit, Medical University of Vienna, Vienna, Austria

^29^Department of Obstetrics and Gynecology, Copenhagen University Hospital, Herlev, Denmark

^30^Maternite Port-Royal, Paris, France

^31^Institute of Cardiovascular and Medical Sciences, British Heart Foundation Glasgow Cardiovascular Research Centre, University of Glasgow, University of Glasgow, Scotland, UK

^32^Section on Islet Cell and Regenerative Biology, Joslin Diabetes Center, Harvard Medical Center, Boston, MA, USA

^33^Northwestern University Feinburg School of Medicine, Northwestern University, Chicago, IL, USA

^34^Manchester University Hospitals, Manchester, UK

^35^Metabolic Unit, Royal Victoria Hospital, Belfast, N. Ireland, UK

^36^Department of Endocrinology and Obstetric Medicine, Mater Health Services and The University of Queensland, South Brisbane, QLD, Australia

^37^Perinatology and Human Reproduction, University of Florence, Florence, Italy

^38^McGill University Health Centre, McGill University, Montreal, QC, Canada

^39^Center of Endocrinology, Northwestern University, Chicago, IL, USA

^40^Department of Medicine, University of East Anglia, Norwich, UK

^41^Maternal & Fetal Health Research Centre, St Mary’s Hospital for Women and Children, Manchester, UK

^42^Diabetes Center, Ebina General Hospital, Kanagawa, Japan

^43^Department of Obstetrics and Gynaecology, Aarhus University Hospital, Aarhus, Denmark

^44^Department of Medicine, Clinical Epidemiology, Karolinska University Hospital, Solna, Sweden

^45^Steno Diabetes Center Copenhagen, Gentofte, Denmark

^46^National Women’s Hospital, Auckland City Hospital, Auckland, New Zealand

^47^Endocrinology, Diabetes and Metabolism, Hospitais da Universidade de Coimbra, Coimbra, Portugal

^48^Division of Maternal-Fetal Medicine, Department of Obstetrics and Gynecology, University of Southern California, Keck School of Medicine, Los Angeles, CA, USA

^49^Department of Obstetrics and Gynaecology, University of Malta Medical School, Mater Dei Hospital, Malta

^50^Leeds Institute of Cardiovascular and Metabolic Medicine, University of Leeds, Leeds, UK

^51^School of Medicine, University of Western Sydney, Campbelltown, NSW, Australia

^52^Department of Obstetrics and Gynecology, Ehime University Graduate School of Medicine, Ehime, Japan

^53^Diabetes Unit, Faculty of Medicine, Semmelweis University, Budapest, Hungary

^54^Department of Gynecology and Obstetrics, Odense University Hospital, Faculty of Health Science, University of Southern Denmark, Odense, Denmark

^55^Department of Diabetes and Endocrinology, Norfolk and Norwich University Hospital NHS Trust, Norwich, UK

^56^Department of Endocrinology and Diabetology, University of Lille, Lille, France

^57^Institute of Sport Science, University of Graz, Graz, Austria

^58^Division of Reproduction, Department of Obstetrics, Gynecology and Gynecological Oncology, Poznan University of Medical Sciences, Poznan, Poland

^59^Athens Diabetes Center, Hygeia General Hospital, Athens, Greece

## Abbreviations

CGM: Continuous glucose monitoring
CSII: Continuous subcutaneous insulin infusion
DPSG: Diabetic Pregnancy Study Group
GDM: Gestational diabetes mellitus
GWG: Gestational weight gain
IADPSG: International Association of Diabetes in Pregnancy Study Groups
LGA: Large for gestational age
NPID: National Pregnancy in Diabetes
SGA: Small for gestational age
